# CYLD links the TRAF6/sNASP axis to TLR4 signaling in sepsis-induced acute lung injury

**DOI:** 10.1007/s00018-025-05654-4

**Published:** 2025-03-20

**Authors:** Yu-Chih Wu, Bing-Hua Su, Wun-Hao Cheng, Cheng-Tai Zou, Edward T. H. Yeh, Feng-Ming Yang

**Affiliations:** 1 School of Respiratory Therapy, College of Medicine, Taipei Medical University, Taipei, 110 Taiwan; 2 Department of Internal Medicine, University of Arkansas for Medical Sciences, Little Rock, AR 72205 USA; 3 Winthrop P. Rockefeller Cancer Institute, University of Arkansas for Medical Sciences, Little Rock, AR 72205 USA

**Keywords:** CYLD, TRAF6, SNASP, TLR4, Sepsis, Acute lung injury

## Abstract

Sepsis-induced acute lung injury (ALI) involves severe lung dysfunction and leads to high morbidity and mortality rates due to the lack of effective treatments. The somatic nuclear autoantigenic sperm protein (sNASP)/tumor necrosis factor receptor-associated factor 6 (TRAF6) axis plays a crucial role in regulating inflammatory responses during sepsis through Toll-like receptor 4 (TLR4) signaling. However, it is unclear whether deubiquitinating enzymes affect the TRAF6/sNASP axis. In this study, we showed that cylindromatosis (CYLD) directly binds to the sNASP and prevents TRAF6 activation. When TLR4 is activated, phosphorylation of sNASP releases CYLD from the TRAF6/sNASP complex, leading to TRAF6 autoubiquitination and the production of proinflammatory cytokines. To stop TRAF6 activation, a complex of sNASP, TRAF6, and CYLD is reformed once dephosphorylation of sNASP occurs by protein phosphatase 4 (PP4). Silencing sNASP negated the inhibitory effects of CYLD on interleukin (IL)-6 and TNF-α production after lipopolysaccharide (LPS) treatment. Similarly, the absence of CYLD also reduced PP4’s negatively regulated production of proinflammatory cytokines, indicating that phosphorylation is crucial for the interaction between sNASP and CYLD as well as TRAF6 activation. Finally, mice infected with a recombinant adenovirus carrying the *CYLD* gene (Ad-CYLD WT), but not a mutation, showed significant reductions in cecal ligation and puncture (CLP)-mediated lung injury and proinflammatory cytokine production. In conclusion, CYLD alleviated sepsis-induced inflammation by interacting with the TRAF6/sNASP axis. These findings suggest that CYLD could be a potential therapeutic target for treating sepsis-induced ALI.

## Introduction

Sepsis is life-threatening multiple organ failure caused by a systemic inflammatory response of the body in response to bacteria, viruses, and other pathogenic microorganisms [1]. According to a global study of adult patients hospitalized for sepsis, the overall mortality rate was 26.7%, while the mortality rate in intensive care units (ICUs) was 41.9% [2]. The high mortality rate and high hospitalization rate due to this disease pose a significant burden to the global economy [3]. During the process of sepsis, the lungs are considered the earliest and most vulnerable target organ, which often results in acute lung injury (ALI). Research indicated that patients with sepsis-induced ALI have weakened gas exchange due to lung inflammation, which leads to pulmonary edema and respiratory failure [4]. Pathological processes include disruption of the alveolar barrier function, pulmonary vascular endothelial damage, and excessive leakage of fluid from pulmonary capillaries into the alveolar space [5]. Many studies found that expressions of inflammatory factors are critical for exacerbating pulmonary edema and promoting ALI [6, 7]. Therefore, controlling alveolar inflammation is important for preventing the occurrence and severity of ALI.

Lipopolysaccharide (LPS), a major component of the outer membrane of gram-negative bacteria, may cause severe inflammatory responses leading to lung injury through interacting with Toll-like receptor 4 (TLR4), which leads to tumor necrosis factor (TNF) receptor-associated factor 6 (TRAF6) activation as well as the nuclear factor (NF)-κB signal pathway which regulates the production of proinflammatory cytokines [8]. Our previous work demonstrated that the binding of somatic nuclear autoantigenic sperm protein (sNASP) to TRAF6 is a critical step in inhibiting TRAF6 activation. In response to LPS, sNASP undergoes phosphorylation, leading to the release of TRAF6, which subsequently promotes TRAF6 autoubiquitination and the activation of downstream kinases [9]. To terminate TLR4 signaling and avoid harmful inflammatory responses, protein phosphatase 4 (PP4) is recruited to dephosphorylate sNASP and then turn off TRAF6 signaling to maintain immune homeostasis [10]. Animal studies also indicated that inhibition of TRAF6 in dominant-negative sNASP mutant mice and a PP4-overexpressing mice model both improved symptoms and systemic inflammation of sepsis-induced ALI [9, 10]. Furthermore, a cell-permeable PEP-sNASP peptide was found to alleviate ALI by targeting TRAF6 autoubiquitination [11]. Taken together, it was suggested that TRAF6 signaling is most closely related to ALI caused by sepsis. However, the molecular mechanisms of sNASP in regulating TRAF6 activation and inflammatory lung injury remain unclear, since no deubiquitylating activity has been detected for sNASP.

Of the various types of posttranslational modifications, the ubiquitination and deubiquitination of host signaling molecules are particularly important in tightly regulating immune and inflammatory responses [12]. Deubiquitinase cylindromatosis (CYLD) exerts a capacity to remove polyubiquitin chains from specific substrates, including TRAF2, TRAF6, TRAF7, and NF-κB essential modulator (NEMO), subsequently restricting TLR signal transduction [13, 14]. CYLD also acts as a negative regulator of mitogen-activated protein kinases (MAPKs), such as p38 MAPK [15, 16]. CYLD is involved in multiple physiological processes, such as immune responses, inflammation, germ cell apoptosis, osteoclastogenesis, and the cell cycle [14, 17, 18]. CYLD dysregulation was linked to the progression of lung injury, cardiovascular diseases, and tumorigenesis [19–21]. Despite the previous identification of CYLD as a lysine 63 (K63)-linked E3 ubiquitin ligase of TRAF6, the molecular mechanism of CYLD underlying the TRAF6/sNASP axis in ALI remains largely unknown.

In this study, we investigated the interplay between CYLD and the TRAF6/sNASP axis, and uncovered a novel functional mechanism of CYLD: that CYLD undergoes separation from the sNASP/TRAF6 complex in sepsis-induced lung innate immunity, which is essential for subsequent TRAF6 autoubiquitination and NF-κB activation. We further provide evidence to show that CYLD inhibits ubiquitinating TRAF-6 probably through directly interacting with sNASP. In vivo, mice infected with a recombinant adenovirus carrying a gene encoding CYLD wild-type (Ad-CYLD WT), but not a mutation, showed markedly attenuated sepsis-induced ALI, with significant reductions in the production of proinflammatory cytokines. Therefore, our study discovered a new anti-inflammatory mechanism of CYLD in sepsis-induced lung inflammatory responses and identified the CYLD-TRAF6/sNASP axis as a potential therapeutic target for treating sepsis-induced ALI.

## Methods

### Cell cultures, plasmids, antibodies, and reagents

HEK293, THP-1 cells, and primary bone marrow-derived macrophages (BMDMs), were maintained as previously described [9]. For THP-1 differentiation, 100 nM phorbol-12-myristate 13-acetate (PMA) (SI-P8139, Sigma-Aldrich, St. Louis, MO, USA) was added for 3 days to obtain phenotypic characteristics of macrophages. Plasmids encoding human HA-CYLD WT and a catalytically inactive CYLD mutant (HA-CYLD mut, amino acids 1–932) were gifts from Shao-Cong Sun (University of Texas MD Anderson Cancer Center, Houston, TX, USA) [22, 23]. Expression plasmids for green fluorescent protein (GFP)-sNASP, Flag-TRAF6, Hemagglutinin (HA)-PP4, and HA-ub, were described previously [9, 10]. Adenoviruses expressing a control vector (Ad-PGK), HA-CYLD (Ad-CYLD WT), and HA-CYLD mutation (Ad-CYLD mut) were generated by the Adenovirus Laboratory at the Institute of Biomedical Sciences in Academia Sinica (Taipei, Taiwan) as previously described [24].

The following antibodies and recombinant proteins were utilized: CYLD (SC-74435, Santa Cruz, Dallas, TX, USA), A20 (SC-166692, Santa Cruz), NASP (SC-161915, Santa Cruz), TRAF6 (SC-7221, Santa Cruz), ubiquitin (SC-8017, Santa Cruz), anti-PPX (SC-374106, Santa Cruz), anti-β-actin (A1544, Sigma-Aldrich), GFP (632,281, Clontech, San Jose, CA, USA), phosphoserine (ab17465, Abcam, Cambridge, UK), HA (H9658, Sigma-Aldrich), Flag (F1804, Sigma-Aldrich), and recombinant human CYLD protein (TP304099, Origene, Rockville, MD, USA). Antibody dilutions were according to the manufacturer’s instructions. LPS (L2630) was purchased from Sigma-Aldrich.

### RNA interference and plasmid transfection

Sequences of small interfering (si)RNAs are listed as follows: siRNAs specific for the genes encoding human *NASP* (siNASP, 5′-GGAACUGCUACCCGAAAUU-3′), *PP4* (siPP4, 5′-CUGGUCGCUUACAUCACUUUA-3′), *TRAF6* (siTRAF6, 5′-CCATGGCAGACGATGATCCC-3′), and *CYLD* (siCYLD, 5′-AAGUACCGAAGGGAAGUAUAG-3′). All siRNAs were synthesized by Genomics (Taipei, Taiwan). As previously outlined [10], cells were exposed to either non-targeting or targeting siRNA using the DharmaFECT 1 reagent (T-2001-02, Horizon, Cambridge, UK) according to the manufacturer’s instructions and harvested 3 days post-transfection. THP-1 cells were transfected using X-tremeGene HP (06–365-752-001, Roche, Basel, Switzerland). Transfection of HEK293 cells was performed using Lipofectamine 3000 (L3000015, Invitrogen, Waltham, MA, USA) according to the manufacturer’s protocol.

### Immunoprecipitation (IP) and immunoblotting (IB)

Harvested cells were subject to IP, followed by resolution of lysates on sodium dodecylsulfate polyacrylamide gel electrophoresis (SDS-PAGE) and a subsequent Western blot analysis, as previously described [9].

### In vitro binding assay

The recombinant GFP-sNASP protein, prepared via IP using GFP-Trap_M (gtm-20, Chromotek, Planegg, Germany), was in vitro phosphorylated by casein kinase II (P6010, New England Biolabs, Ipswich, MA, USA) for 60 min at 30 °C. Subsequently, it was incubated with the indicated recombinant protein for an additional 120 min at 4 °C. Samples were then subjected to IP and IB using the indicated antibodies.

### Real-time polymerase chain reaction (PCR)

RNA samples were extracted using the Toolsmart RNA extractor (TB-DPT-BD24, Biotools, Taipei, Taiwan), and 1 µg of total RNA was utilized for the real-time PCR analysis with a magic RT cDNA synthesis kit (Bio-Genesis Technologies, Taipei, Taiwan). SYBR Green Master Mix (ThermoFisher Scientific, Waltham, MA, USA) was used for the real-time PCR, and fluorescence was monitored using an StepOnePlusTM machine (ThermoFisher Scientific). Below are the primer sequences: human IL-6 (forward 5′-TACATCCTCGACGGCATCT-3′ and reverse 5′-ACCAGGCAAGTCTCCTCAT-3′); and human TNF-α (forward 5′-TGGAGCTGGCCGAGGAG-3′ and reverse 5′-AGCAGGCAGAAGAGCGTGG-3′.

### Cecal ligation and puncture (CLP) animal model

Animal protocols were approved by the Animal and Ethics Review Committee of the Laboratory Animal Center at Taipei Medical University. Eight-week-old male C57BL/6JNarl mice were obtained from the National Laboratory Animal Center (Taipei, Taiwan).

Recombinant adenoviruses expressing a control vector (Ad-PGK), HA-CYLD wild-type (Ad-CYLD WT), and HA-CYLD mutant (Ad-CYLD mut) were generated by the Adenovirus Laboratory at the Institute of Biomedical Sciences, Academia Sinica (Taipei, Taiwan), as previously described [24].

For animal experiments involving adenoviruses, mice were intranasally injected with recombinant adenovirus (0.1–0.5 × 10⁹ pfu per mouse). Three days post-injection, a CLP-induced polymicrobial sepsis model was established using aseptic surgical techniques, as described previously [25]. After 24 h, mice were euthanized, and lung samples were collected for hematoxylin and eosin (H&E) staining and RT-PCR analysis. Serum was also collected to assess cytokine expression.

### Enzyme-linked immunosorbent assay (ELISA)

Concentrations of cytokines in cell culture supernatants were assessed using the ELISA MAX™ Deluxe Set Human IL-6 (#430504) and TNF-α (#430204) from BioLegend (San Diego, CA, USA), following the manufacturer’s instructions.

### shRNA knockdown

The shRNA-expressing lentiviral plasmids (pLKO.1-shRNA) were obtained from National RNAi Core Facility (Academia Sinica, Taipei, Taiwan). NASP was efficiently targeted with construct TRCN0000099303, and PP4 was targeted with the mixture of construct TRCN0000080833, TRCN0000080835 and TRCN0000080836. The shRNA construct (TRCN0000072223) targeting the LacZ was used as a control. Lentiviral particles were prepared as described previously [26].

### Statistical analysis

Statistical analysis was conducted using GraphPad Prism 6 software (GraphPad Software, Boston, MA, USA). Student’s *t*-test was utilized for comparisons between two groups, while a one-way analysis of variance (ANOVA) was employed for comparisons involving three or more groups. Values with a significance level of *p* < 0.05 were deemed statistically significant.

## Results

### CYLD directly interacts with the sNASP

**Fig. 1:**
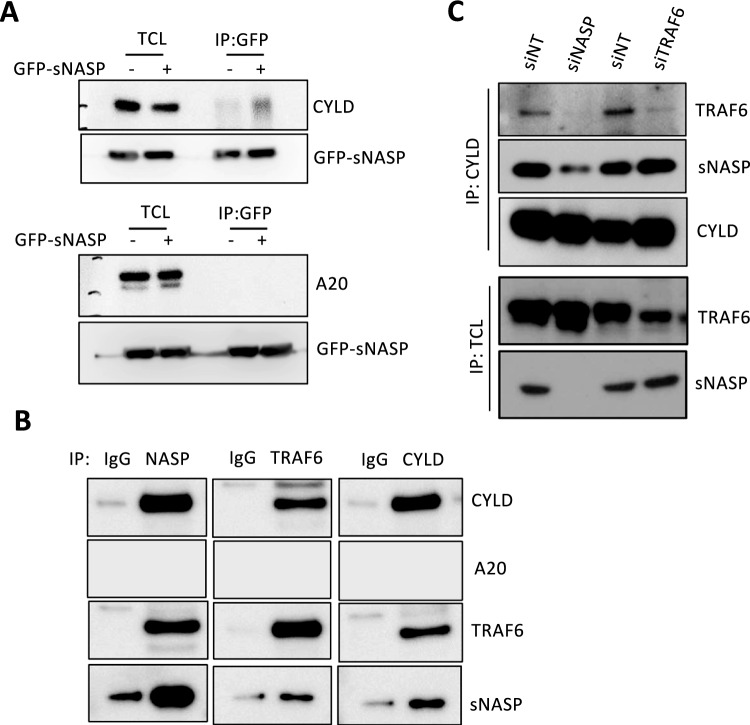
Deubiquitinase CYLD interact TRAF6/sNASP complex. **A** Immunoprecipitation (IP) of GFP-sNASP (with anti-GFP) from HEK293 cells transiently transfected with GFP-sNASP ( +) or empty vector (−), followed by immunoblotting (IB) with antibodies against GFP, A20 or CYLD. TCL, total cell lysates. **B** IP of endogenous TRAF6, sNASP, CYLD or IgG from THP-1 cells, followed by IB with indicated antibodies. **C** IP of endogenous CYLD from THP-1 cells transfected with siRNA negative control (siNT), siNASP or siTRAF6, followed by IB with antibodies against TRAF6, NASP or CYLD. TCL IB was done with anti-TRAF6 or anti-NASP. *n* = 3

The sNASP was shown in our previous publication to negatively regulate TLR4-induced proinflammatory cytokine production through inhibition of TRAF6 autoubiquitination [9, 11]. Since we failed to detect any deubiquitinylating activity of sNASP, we questioned whether deubiquitinating enzymes (DUBs) are involved in sNASP’s inhibition of the ubiquitination of TRAF6. A previous report indicated that A20 and CYLD, two DUBs, were involved in the control of TRAF6 ubiquitination [27]. Herein, we demonstrated that GFP-sNASP binds to CYLD, not A20, in HEK293 cells (Fig. 1A). Similarly, endogenous CYLD interacted with sNASP or TRAF6 in THP-1 cells (Fig. 1B), suggesting that CYLD directly binds to the TRAF6/sNASP complex. Silencing sNASP completely annulled the interaction between CYLD and TRAF6. Otherwise, silencing TRAF6 did not change the interaction between sNASP and CYLD (Fig. 1C), suggesting that sNASP is a critical adaptor for the formation of a complex consisting of CYLD and TRAF6.

### CYLD inhibits TRAF6 polyubiquitination through sNASP

**Fig. 2:**
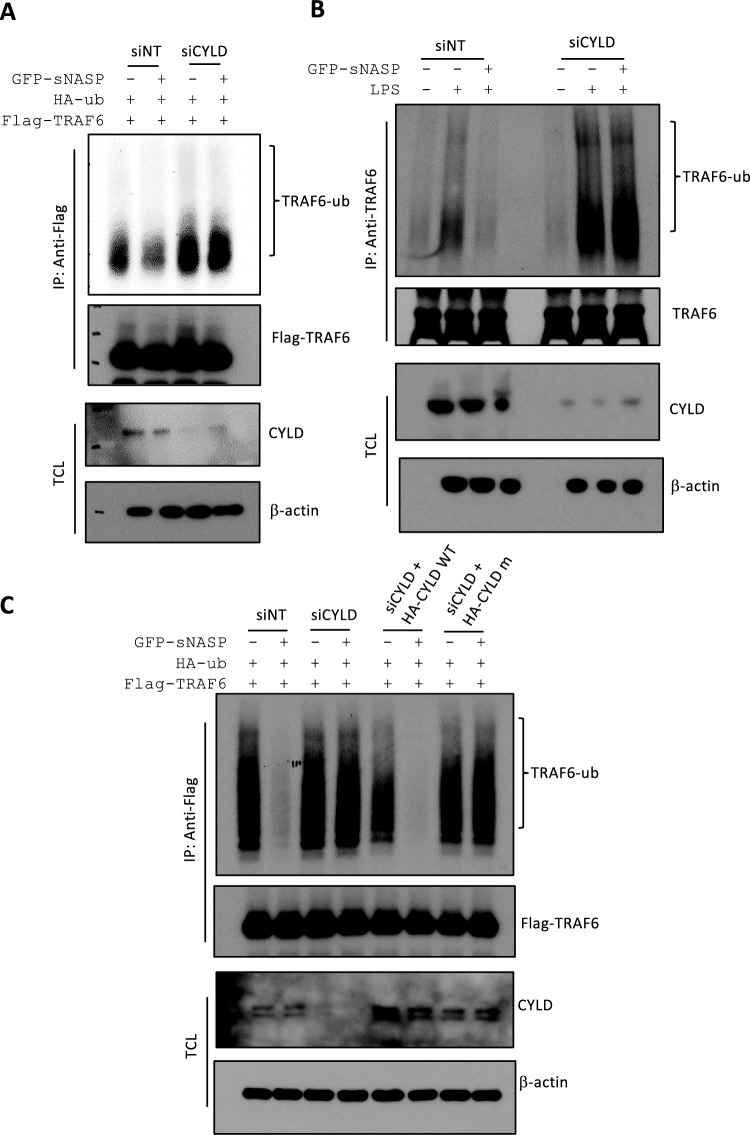
CYLD inhibited TRAF6 ubiquitination through sNASP in a resting status. **A** Immunoprecipitation (IP) of Flag-TRAF6 (with anti-Flag agarose) from HEK293 cells transfected with siNT or siCYLD in the presence ( +) or absence (−) of GFP-tagged sNASP, Flag-tagged TRAF6 or HA-ub, followed by immunoblotting (IB) with antibody against Flag or HA. TCL IB was done with anti-CYLD and β-actin. **B** THP-1 cells were transfected with siRNA negative control (siNT) or siCYLD in the presence ( +) or absence (−) of GFP-tagged sNASP, stimulated with LPS (1 µg/ml) for 1 h and followed by IB with antibodies against TRAF6 or ub after IP with anti-TRAF6. TCL IB was done with anti-CYLD or anti-β-actin. **C** IP of Flag-TRAF6 (with anti-Flag agarose) from HEK293 cells transfected with siNT, siCYLD, siCYLD plus HA-tagged CYLD WT, or siCYLD plus HA-tagged CYLD mutant in the presence ( +) or absence (−) of Flag-tagged TRAF6, HA-tagged ub or GFP-tagged sNASP, followed by IB with antibodies against HA or Flag. TCL IB was done with anti-CYLD and β-actin. *n* = 3

Since our previous report showed sNASP to be a negative regulator of TRAF6 signaling [9], we speculated that CYLD was involved in sNASP's downregulation of TRAF6 poly-ubiquitination. First, we examined the level of TRAF6 ubiquitination in siCYLD-treated cells. TRAF6 ubiquitination increased when CYLD was downregulated, and depletion of CYLD also abolished the effect of sNASP on reducing TRAF6 autoubiquitination (Fig. 2A, B). Next, overexpression of CYLD WT, but not the mutant, restored sNASP's function of negatively regulating TRAF6 polyubiquitination in siCYLD-treated cells compared to siNT (Fig. 2C). These results showed a new mechanism of sNASP bringing TRAF6 and CYLD together to regulate TRAF6 polyubiquitination.

### CYLD negatively regulates proinflammatory cytokine responses mediated by sNASP

**Fig. 3:**
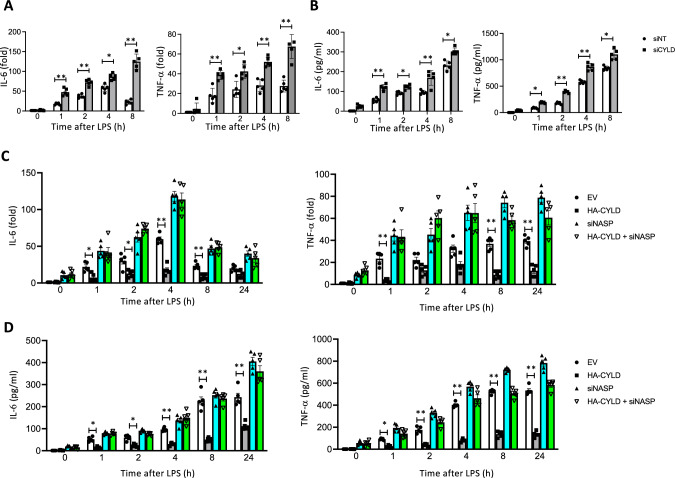
CYLD negatively regulates proinflammatory cytokine production through sNASP. **A** and** C** RNA expression level of TNF-α and IL-6 in THP-1 cells transduced with (A) siNT or siCYLD, or (C) empty vector (EV), HA-tagged CYLD with or without siNASP, and stimulated with LPS (1 µg/ml). Results were normalized to the expression of ACTB (encoding β-actin) and are presented relative to those of untreated cells. **B** and** D** Production of TNF-α and IL-6 by THP-1 cells transduced as in A and C and stimulated with LPS (1 µg/ml). Data are the mean ± SE for each group. *p < 0.05, **p < 0.01 (by a one-way ANOVA). *n* = 5

To investigate CYLD's role in negatively regulating TLR4 signaling activation, we conducted a real-time PCR and an ELISA. Silencing CYLD significantly boosted LPS-induced IL-6 and TNF-α expressions at both the messenger (m)RNA and protein levels (Fig. 3A, B). Next, we transfected cells with a CYLD-expressing plasmid, either with or without siRNA targeting NASP (siNASP), and then stimulated these cells with LPS (1 µg/ml). Overexpressing CYLD led to significant decreases in LPS-induced TNF-α and IL-6, at both the transcription and translation levels, compared to an empty vector (EV) (Fig. 3C, D). However, when sNASP was downregulated, the effects of CYLD on TNF-α and IL-6 production were abolished (Fig. 3C, D), indicating that sNASP is essential for CYLD-mediated suppression of LPS-induced proinflammatory cytokines. These findings indicated that CYLD negatively regulates activation of TLR4-triggered proinflammatory cytokine responses through sNASP.

### Dephosphorylation of sNASP by PP4 is necessary for formation of the sNASP/TRAF6/CYLD complex

**Fig. 4:**
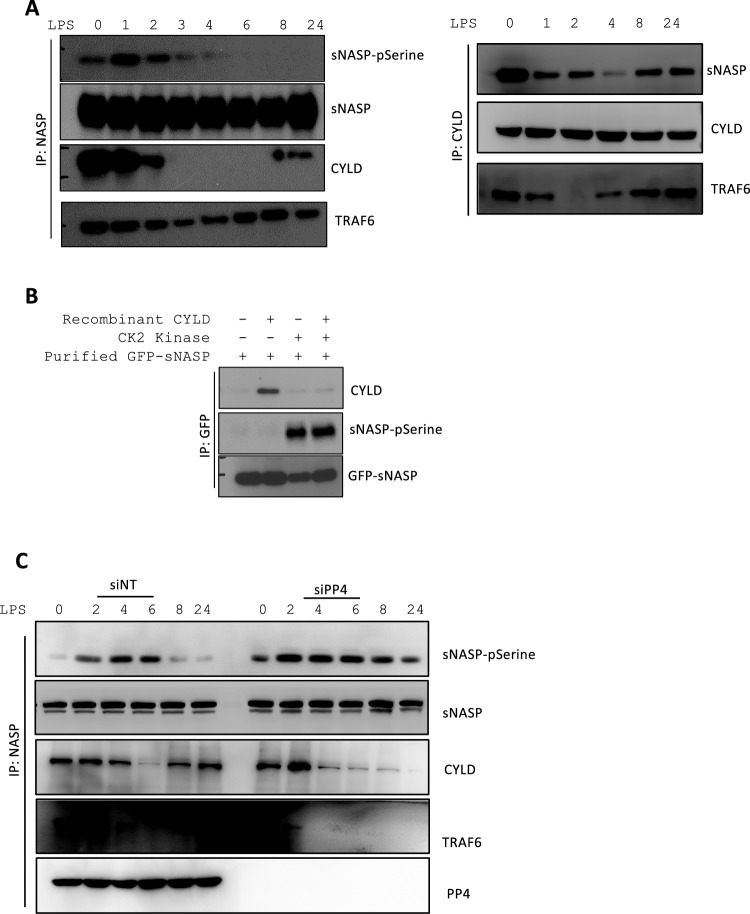
Dephosphorylation of sNASP by PP4 is required for the sNASP/TRAF6/CYLD complex formation. **A** THP-1 cells were stimulated with LPS for different time points and and assessed by IB with indicated antibodies after IP with anti-NASP or anti-CYLD. **B** The Purified GFP-tagged sNASP protein was incubated with CK2 kinase with or without recombinant CYLD protein. The reaction products were followed by IB with antibodies against phosphorylated serine (pSerine), GFP or CYLD after IP with anti-GFP. **C** THP-1 cells were transfected with siRNA negative control (siNT) or siPP4, followed by IB with antibodies against phosphorylated serine (pSerine), TRAF6, sNASP, or CYLD after IP with anti-sNASP. TCL IB was done with anti-PP4 or anti-β-actin. *n* = 3

Next, we explored whether phosphorylation changed formation of the sNASP/TRAF6/CYLD complex during TLR4 signaling. IP assays revealed that endogenous CYLD was associated with sNASP and TRAF6 in unstimulated cells (Fig. 4A). One hour after LPS treatment, sNASP showed serine phosphorylation in THP-1 cells, which is consistent with our previous discovery [9]. Reciprocal IP and Western blot analyses confirmed that the dissociation of endogenous CYLD from sNASP was correlated with increased serine-specific phosphorylation (Fig. 4A), which suggests that serine phosphorylation may indeed play a role in regulating the interaction between sNASP and CYLD. These findings were consistent with results from an in vitro assay. In this assay, the CYLD recombinant protein was incubated with purified GFP-tagged sNASP in the presence or absence of the casein kinase 2 (CK2) kinase. Phosphorylation of sNASP increased in the presence of the CK2 kinase. The CYLD recombinant protein only exhibited an interaction with GFP-tagged sNASP in the absence of the CK2 kinase (Fig. 4B). These results suggest that CYLD can directly interact with non-phosphorylated sNASP during TLR4 signaling.

We previously demonstrated that PP4 is recruited by phosphorylated sNASP after LPS stimulation to form a sNASP/TRAF6 complex and subsequently inhibits TRAF6 autoubiquitination, which leads to termination of TLR4 signaling. Thus, we questioned whether PP4 is involved in homeostasis of the CYLD/sNASP/TRAF6 complex during TLR4 signaling. THP-1 cells were transfected with or without siPP4 followed by LPS. In the siNT control, dissociation of the CYLD/sNASP/TRAF6 complex was observed from 2 to 4 h following LPS stimulation, and formation of the complex had been restored by 8 h following LPS stimulation. PP4-knockdown completely abolished restoration of the CYLD/sNASP/TRAF6 complex (Fig. 4C). This suggests that silencing PP4 prolonged the phosphorylation of sNASP which impeded formation of the CYLD/sNASP/TRAF6 complex. Thus, our data suggested that PP4 is also critical for homeostasis of the CYLD/sNASP/TRAF6 complex.

### Involvement of CYLD in PP4 inhibits TRAF6 polyubiquitination

**Fig. 5:**
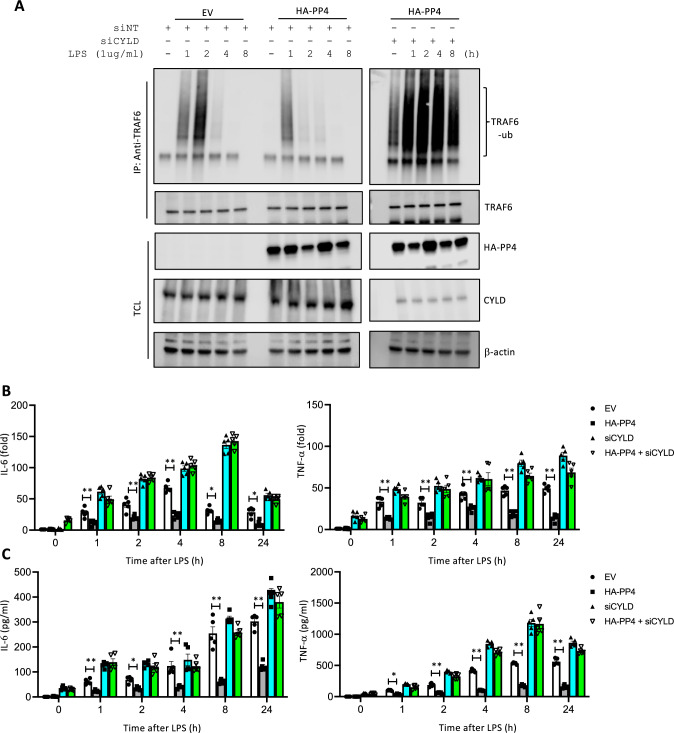
Loss of CYLD ameliorate PP4’s negatively regulated proinflammatory cytokines production. **A** IP of TRAF6 (with anti-TRAF6) from THP-1 cells transfected with empty vector (EV) or HA-PP4 in the presence ( +) or absence (−) of siNT or siCYLD followed by IB with antibodies against ub, TRAF6 or HA. TCL IB was done with anti-CYLD and β-actin. **B** RNA expression level of TNF-α and IL-6 in THP-1 cells transduced with the EV, HA-tagged PP4 with or without siCYLD, and stimulated with LPS. Results were normalized to the expression of ACTB (encoding β-actin) and are presented relative to those of untreated cells. **C** Production of TNF-α and IL-6 by THP-1 cells transduced as in Band stimulated with LPS. Data are the mean ± SE for each group. *p < 0.05, **p < 0.01 (by a one-way ANOVA). *n* = 5

Since dephosphorylation of sNASP by PP4 regulates interactions between TRAF6 and sNASP, we asked whether PP4 is also involved in CYLD’s negative regulation of TRAF6 polyubiquitination. Overexpression of PP4 significantly reduced autoubiquitination of TRAF6 in a time-dependent manner following LPS stimulation. However, loss of CYLD increased TRAF6 polyubiquitination in PP4-overexpressing THP-1 cells (Fig. 5A). We also examined proinflammatory cytokines through a real-time PCR and ELISA using transfection with a PP4-expressing plasmid in the absence or presence of siCYLD and LPS stimulation. Consistently, PP4 overexpression resulted in dramatic losses of LPS-induced TNF-α and IL-6 at both the mRNA and protein levels, compared to an EV (Fig. 5B, C). However, siCYLD completely abrogated the effect of PP4 on TNF-α and IL-6 production (Fig. 5B, C), suggesting that synergy between CYLD and PP4 is critical for TLR4-mediated proinflammatory cytokine expressions.

### CYLD interacted with the sNASP/TRAF6 complex and negatively regulated proinflammatory cytokines in primary bone marrow-derived macrophages (BMDMs)

**Fig. 6:**
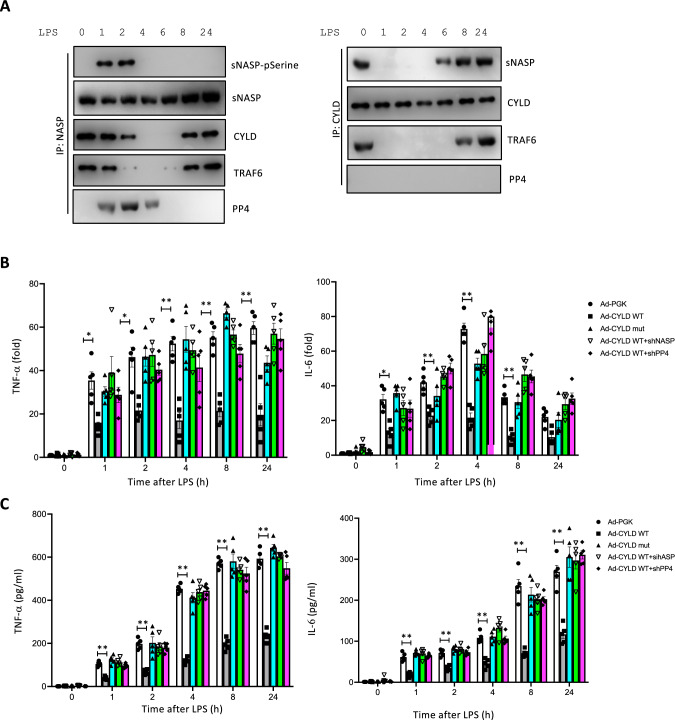
CYLD directly interacts with the sNASP/TRAF6 complex and negatively regulates proinflammatory cytokines in BMDMs. **A** BMDM cells were stimulated with LPS for different time points and and assessed by IB with indicated antibodies after IP with anti-NASP or anti-CYLD. **B** RNA expression level of TNF-α and IL-6 in BMDM cells infected with adenoviruses expressing control vector (Ad-PGK) or a gene encoding HA-PP4 WT (Ad-PP4 WT) or HA-PP4 mutant (Ad-PP4mut) in the presence or absence of shNASP or shPP4 were stimulated with LPS for different time points. **C** Production of TNF-α and IL-6 by BMDM transduced as in B and stimulated with LPS. Data are the mean ± SE for each group. *p < 0.05, **p < 0.01 (by a one-way ANOVA). *n* = 5

To address the physiologic relevance, primary BMDMs were isolated and used to examine the effects of CYLD in sNASP/TRAF6 interactions and proinflammatory cytokine production. Similar to previous results, IP assays showed that endogenous CYLD had dissociated from the sNASP/TRAF6 complex by 2 h after LPS stimulation, which was correlated with increased serine-specific phosphorylation in BMDMs (Fig. 6A). Reciprocal IP and Western blot analyses further confirmed the LPS-induced dissociation of endogenous CYLD from the sNASP/TRAF6 complex (Fig. 6A). Next, we infected BMDMs with an adenovirus expressing either HA-tagged CYLD WT (Ad-CYLD) or mutant (Ad-CYLDmut) in the presence or absence of a lentivirus expressing shNASP or shPP4. Significant reductions in TNF-α and IL-6 were observed in CYLD WT-overexpressing BMDMs, but not in the CYLD mutant (Fig. 6B, C). However, loss of either sNASP or PP4 completely eliminated the effects of CYLD on TNF-α and IL-6 production (Fig. 6B, C). Taken together, this suggested that both sNASP and PP4 are required for CYLD to suppress LPS-triggered proinflammatory cytokines in human macrophages.

### CYLD alleviated sepsis-induced pathological changes in the lungs

**Fig. 7:**
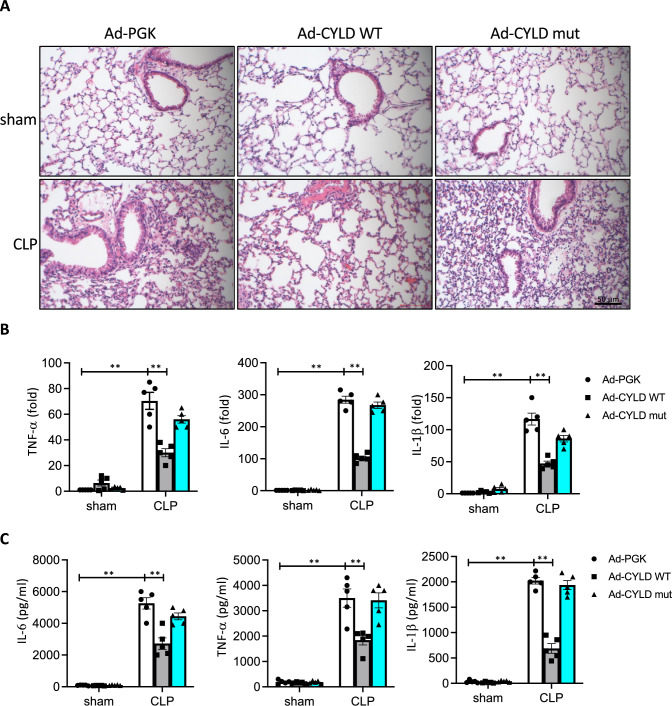
CYLD protect the mice from CLP-induced acute lung injury. B6 mice were intranasally injected with adenoviruses expressing an empty vector (Ad-PGK), HA-CYLD wild-type (Ad-CYLD WT), or HA-CYLD mutant (Ad-CYLD mut) for three days, followed by CLP for 24 h. **A** Lungs from each group of mice were fixed and stained with H&E. Scale bar = 50 μm. **B** RNA expressions of TNF-α, IL-6, and IL-1β in lung tissues were determined by a RT-qPCR. **C** Levels of TNF-α, IL-6, and IL-1β in peripheral blood were detected by an ELISA. *n* = 5, **p < 0.01 (by a one-way ANOVA)

**Fig. 8:**
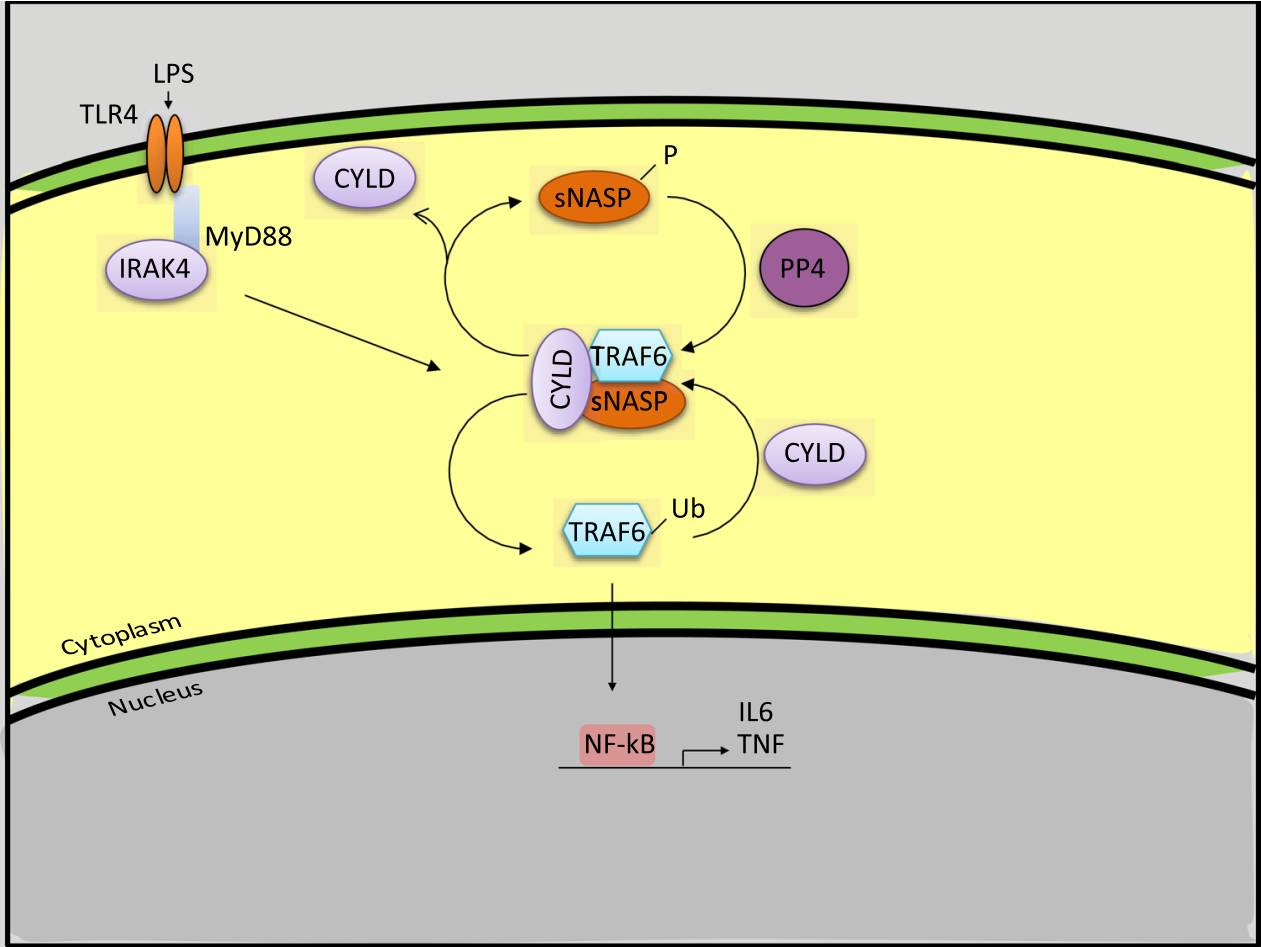
Model of TLR4-mediated sNASP/TRAF6 axis regulated by CYLD. In resting status, CYLD forms a complex with the TRAF6/sNASP complex to maintain immune homeostasis. Upon LPS stimulation, CYLD dissociates from phosphorylated sNASP and then releases TRAF6, which results in TRAF6 autoubiquitination and activation of downstream kinases. To control TRAF6 activation, CYLD again is recruited to the TRAF6/sNASP complex after PP4 dephosphorylation of sANSP to prevent an overwhelming production of proinflammatory cytokines

To verify the functional role of CYLD as an attenuator of LPS stimulation in an animal model, we injected either Ad-CYLD WT or Ad-CYLD mut into a sepsis-induced murine ALI model. First, we evaluated pathological changes in the lungs as detected by H&E staining to assess the effects of CYLD on LPS-induced lung tissue injury. As shown in Fig. 7A, mice lungs challenged with CLP displayed severe inflammatory cell accumulation and destruction of the alveolar histological structure, compared to sham group mice. The pathology of lung injury was markedly decreased in Ad-CYLD WT mice, but not in Ad-CYLD mut mice, indicating that CYPD protected against sepsis-induced lung injury. To further evaluate the anti-inflammatory activity of CYLD, we detected the production of proinflammatory cytokines in the lungs and peripheral blood serum by a quantitative PCR and ELISA, respectively. As shown in Fig. 7B, compared to the control Ad-PGK and Ad-CYLD mut groups, mRNA expressions of TNF-α, IL-6, and IL-1β in lung tissues were significantly reduced in the Ad-CYLD WT group. And protein levels of TNF-α, IL-6, and IL-1β in peripheral blood serum were also decreased by Ad-PP4 WT transduction (Fig. 7C). Overall, our data demonstrated that CYLD significantly attenuated LPS-induced pathological injury and inflammatory activities in the lungs (Fig. 8).

## Discussion

CYLD was reported to be a key modulator of immune responses, inflammation, thymocyte development, B-cell activation, and tumor cell growth [12, 17, 28, 29]. There is compelling evidence of the important roles of CYLD in negatively regulating NF-κB activation. The NF-κB transcription factor, which can be activated by both gram-negative and gram-positive bacterial pathogens or their products, is essential for inducing CYLD. In turn, CYLD negatively regulates NF-κB signaling induced by these bacteria [30, 31]. For instance, CYLD negatively regulates *Streptococcus pneumoniae-* and *Escherichia coli*-induced proinflammatory mediators [20, 32]. CYLD removes ubiquitin from TRAF6 and TRAF7, thereby downregulating TLR2 signaling and inflammation triggered by peptidoglycan [27]. The absence of CYLD protects mice from ALI caused by lethal *Streptococcus pneumoniae* infections by suppressing expression of plasminogen activator inhibitor (PAI)−1 [19, 33]. Furthermore, evidence indicates that CYLD negatively regulates the *Streptococcus pneumoniae*-induced nuclear factor of activated T cells (NFAT) signaling pathway by deubiquitinating transforming growth factor (TGF)-β-activated kinase 1 (TAK1) [32]. In contrast, CYLD-deficient mice show increased susceptibility to *Escherichia coli* pneumonia, accompanied by enhanced NF-κB activation [20]. Herein, our in vitro data demonstrated that CYLD expression negatively regulates TRAF6/sNASP axis-dependent proinflammatory cytokines. In contrast, the loss of CYLD led to significant TRAF6 activation, and promoted TNF-α and IL-6 production. Those in vivo results were confirmed by CLP-challenged mice with CYLD expression, which markedly alleviated ALI in mice following CLP. These discoveries further confirmed that CYLD might serve as a potential drug target for treating sepsis-induced pneumonia.

TRAF6 plays a pivotal role in innate and adaptive immunity, and tight regulation of its signaling activity is crucial for maintaining immunological homeostasis [34]. Our previous study was the first to reveal that sNASP negatively regulates TRAF6 autoubiquitination in response to LPS signaling. Additionally, we identified the mechanism by which CK2 phosphorylation of sNASP releases it from TRAF6, thereby promoting proinflammatory signaling [9]. How sNASP negatively regulates TRAF6 signaling has remained poorly understood, although this function of sNASP is known to require its N-terminal region [11]. The biological function of sNASP was initially shown to be a histone chaperone that binds core histones (H3 and H4) and linker histones (H1), transports linker histones into the nucleus, and transfers H1 histones onto DNA to facilitate chromatin assembly [35, 36]. Previous research showed that TRAF6 activation relies on the K63 auto-polyubiquitination process, which can be inhibited by deubiquitinases such as CYLD, A20, ubiquitin-specific protease 4 (USP4), USP2a, or monocyte chemoattractant protein (MCP)-induced protein 1 [37–41]. Our present study demonstrates that sNASP physically interacts with CYLD, rather than A20, and facilitates CYLD binding to TRAF6. Under immune homeostasis, CYLD is integrated into the TRAF6/sNASP complex, playing a crucial role in preventing spontaneous TRAF6 activation and subsequent inflammatory signaling through this mechanism. Upon LPS stimulation, CYLD and sNASP were separated which allowed TRAF6 to oligomerize and fully activate. As the inflammatory response progresses, the reassembled CYLD/sNASP/TRAF6 complex may act to terminate TRAF6 activity and inflammatory action. These findings suggest an intriguing molecular interplay between CYLD and the TRAF6/sNASP complex and shed light on how sNASP as a critical adaptor protein negatively regulates TRAF6 signaling.

Phosphorylation can be used to modulate the strength of protein–protein interactions, thereby regulating protein binding and coordinating different signaling pathways [42]. There is compelling evidence that balancing the phosphorylation and dephosphorylation of proteins is crucial for initiating, driving, and terminating TLR signaling [43]. For instance, IL-1 receptor-associated kinase 4 (IRAK4) phosphorylates IRAK1 and then triggers conformational changes in IRAK1, which facilitate IRAK1 being released from the myddosome to activate TRAF6 signaling [44, 45]. On the contrary, PP2A was shown to dephosphorylate IRAK1 and moderate its stability and kinase activity [46]. In our previous discovery, phosphorylation of sNASP by CK2 is pivotal to the release TRAF6 from the TRAF6/sNASP complex and promotes downstream signaling pathways. Dephosphorylation by PP4 can reverse and limit TLR pathway output. Herein, we further demonstrated that CYLD directly binds to sNASP, not TRAF6, since loss of TRAF6 did not change the interaction between CYLD and sNASP. Upon LPS stimulation, CYLD also dissociated from phosphorylated-sNASP, allowing TRAF6 activation. Moreover, TRAF6 polyubiquitination and proinflammatory cytokine production were not suppressed in sNASP-deficient cells by CYLD overexpression, which led us to propose that CYLD cannot inactivate TRAF6 in the absence of sNASP. Similar results were also found in CYLD-deficient cells by the overexpression of PP4. This suggests that CYLD binding to sNASP is critical to negatively regulate TRAF6 signaling, and a balance between sNASP phosphorylation and dephosphorylation is a key modification controlling the interaction. Thus, our work has elucidated an alternative mechanism of CYLD in regulating TLR4/TRAF6 signaling.

Alveolar macrophages are central to modulating inflammation by recruiting neutrophils and circulating macrophages to the site of injury via deubiquitinating enzymes [47, 48]. For example, USP7 and USP47 promote the NLRP3 inflammasome by accelerating an apeck-like protein containing a CARD (ASC) oligomerization in macrophages [49]. In addition, inhibition of UPS25 reduces transcription of inflammatory genes in monocyte THP-1 cells [50]. However, the role of CYLD is still controversial. CYLD is highly induced by *Streptococcus pneumoniae* pneumolysin (PLY). A CYLD deficiency protects mice from ALI in lethal PLY infections by inhibiting plasminogen activator inhibitor (PAI)−1 expression [19, 33]. Additional evidence indicates that CYLD negatively regulates the nuclear factor of activated T cells (NFAT) signaling pathway by deubiquitinating TGF-β-activated kinase 1 (TAK1) in PLY-induced inflammation [32]. In contrast, CYLD-deficient mice exhibit increased susceptibility to *Escherichia coli* pneumonia, accompanied by enhanced NF-κB activation [20]. It is possible that different pathogens may utilize distinct mechanisms to promote lung inflammation. Results of our cellular and animal studies are in line with recent reports which demonstrated that CYLD administration prevented excessive activation of proinflammatory cytokine genes in macrophages.

## Conclusion

In summary, we demonstrated that CYLD binds to the TRAF6/sNASP complex in an unstimulated status. Following LPS stimulation, CYLD was released and allowed TRAF6 to activate downstream NF-κB and proinflammatory cytokine genes. During the late stage of infection, CYLD re-associated with the TRAF6/sNASP complex to terminate infection-induced lung inflammation by inhibiting TRAF6 activation. These discoveries indicated the inhibitory effect of CYLD in sepsis-induced inflammatory responses and may be a potential drug target for treating pneumonia due to bacterial infection.

## Data Availability

All data are reported in the present study.

## Abbreviations

ALI: Acute lung injury
CLP: Cecal ligation and puncture
CYLD: Cylindromatosis
LPS: Lipopolysaccharide
TLR4: Toll-like receptor 4
TRAF6: Tumor necrosis factor receptor-associated factor 6
sNASP: Somatic nuclear autoantigenic sperm protein
